# The double-edged sword of (re)expression of genes by hypomethylating agents: from viral mimicry to exploitation as priming agents for targeted immune checkpoint modulation

**DOI:** 10.1186/s12964-017-0168-z

**Published:** 2017-03-31

**Authors:** Florian Wolff, Michael Leisch, Richard Greil, Angela Risch, Lisa Pleyer

**Affiliations:** 1 Department of Molecular Biology, University of Salzburg, Salzburg, Austria; 2 3rd Medical Department with Hematology and Medical Oncology, Hemostaseology, Rheumatology and Infectious Diseases, Laboratory for Immunological and Molecular Cancer Research, Oncologic Center, Paracelsus Medical University Salzburg, Müllner Hauptstraße 48, A-5020 Salzburg, Austria; 3 Salzburg Cancer Research Institute - Center for Clinical Cancer and Immunology Trials, Salzburg, Austria; 4 Cancer Cluster Salzburg, Salzburg, Austria

**Keywords:** DNA methylation, Tumor microenvironment, Hypomethylating agents, Endogenous retroviral elements, Immune checkpoint blockade, Immune checkpoint inhibitors, Decitabine, Azacitidine, Acute myeloid leukemia, Cancer

## Abstract

Hypomethylating agents (HMAs) have been widely used over the last decade, approved for use in myelodysplastic syndrome (MDS), chronic myelomonocytic leukemia (CMML) and acute myeloid leukemia (AML). The proposed central mechanism of action of HMAs, is the reversal of aberrant methylation in tumor cells, thus reactivating CpG-island promoters and leading to (re)expression of tumor suppressor genes. Recent investigations into the mode of action of azacitidine (AZA) and decitabine (DAC) have revealed new molecular mechanisms that impinge on tumor immunity via induction of an interferon response, through activation of endogenous retroviral elements (ERVs) that are normally epigenetically silenced. Although the global demethylation of DNA by HMAs can induce anti-tumor effects, it can also upregulate the expression of inhibitory immune checkpoint receptors and their ligands, resulting in secondary resistance to HMAs. Recent studies have, however, suggested that this could be exploited to prime or (re)sensitize tumors to immune checkpoint inhibitor therapies. In recent years, immune checkpoints have been targeted by novel therapies, with the aim of (re)activating the host immune system to specifically eliminate malignant cells. Antibodies blocking checkpoint receptors have been FDA-approved for some solid tumors and a plethora of clinical trials testing these and other checkpoint inhibitors are under way. This review will discuss AZA and DAC novel mechanisms of action resulting from the re-expression of pathologically hypermethylated promoters of gene sets that are related to interferon signaling, antigen presentation and inflammation. We also review new insights into the molecular mechanisms of action of transient, low-dose HMAs on various tumor types and discuss the potential of new treatment options and combinations.

## Background

### Introduction to hypomethylating agents (HMAs)

DNA methylation refers to the stable and reversible addition of a methyl group to position 5 of the cytidine ring within cytosine-phosphate-guanine (CpG) dinucleotides in DNA [1]. Methylcytosine has been termed the fifth base [2]. Enzymes that recognize, alter and maintain CpG methylation have been intensively investigated in recent years; and advances in array-based and next-generation sequencing technologies have made it possible to analyze changes in DNA methylation at different stages of disease. Consequently our understanding of CpG methylation and its entanglement with other epigenetic pathways (i.e. histone modifications and short regulatory RNAs), as well as their roles in disease initiation and propagation, has broadened considerably [3, 4].

**Fig. 1:**
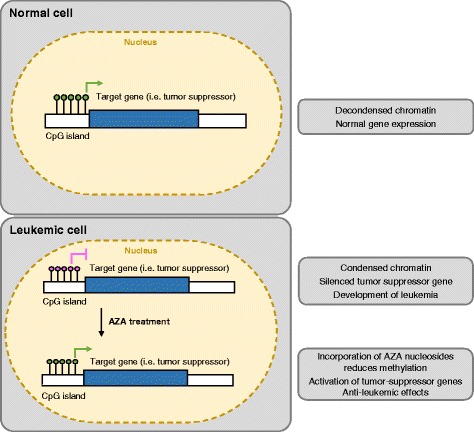
Methylation patterns in MDS/AML and mechanisms of action of AZA and DAC. 1) In normal human cells, CpG islands in the promoter region of tumor suppressor genes are unmethylated (indicated by *green dots*), allowing transcription of these genes. 2) Hypermethylation of tumor suppressor genes (indicated as *red dots*) in the pathogenesis of MDS leads to silencing of tumor suppressor genes and development of a leukemic phenotype. 3) Treatment with AZA nucleosides causes demethylation of the hypermethylated CpG islands in MDS/AML leading to reactivation of tumor suppressor genes and anti-leukemic effects

Global changes in DNA methylation patterns have been linked to the onset and progression of malignant transformation; tumor cells can exhibit aberrant genome-wide hypomethylation and hypermethylation of CpG island promoters [5]. Aberrant hypomethylation supports genome instability and can activate proto-oncogenes [6, 7], whereas hypermethylation of CpG island promoters can silence tumor suppressor genes (TSGs) (Fig. 1) [8]. It has thus been proposed that methylation of genes involved in disease etiopathogenesis may act as biomarkers in several diseases including solid tumors and AML [9–13].

**Fig. 2:**
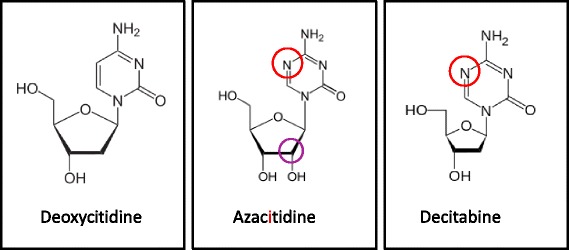
Structure of azanucleosides. Structure of deoxycitidine and the two azanucleosides azacitidine (AZA) and decitabine (DAC). DAC is the 2′didesoxy form of AZA, incorporated into DNA upon triphosphorylation. AZA is primarily incorporated into RNA. Upon triphosphorylation and reduction by the enzyme ribonucleotide reductase it is also incorporated into DNA. The red circles highlight structural differences between deoxycytidine and the two azanucleosides AZA and DAC. The purple circle highlights the structural difference between AZA and DAC

Improved understanding of epigenetic mechanisms in cell biology and tumor pathogenesis has fueled the development of therapies with the primary goal of reversing aberrant epigenetic signaturesand undermining tumor cell immunity. Hypomethylating agents, such as the two nucleoside analogs 2′-deoxy-5-azacitidine/decitabine (DAC) and 5-azacitidine/azacitine (AZA), target the aberrant methylation of DNA to reverse epigenetic silencing and reactivate tumor suppressor genes (TSGs). When given at low doses, DAC and AZA (Fig. 2) induce global demethylation in tumor cells (reviewed in [14]). Global demethylation upon HMA exposure is explained by mechanisms that deplete and/or destabilize the DNA methyltransferase DNMT1 in cells.

DNMT1 is responsible for the maintenance of established DNA methylation patterns on newly synthesized DNA strands during replication. Blocking this enzyme results in passive DNA-replication-dependent demethylation during cell division. Upon triphosphorylation by cytosolic kinases, DAC is directly incorporated into DNA during the S-phase of the cell cycle whereas AZA is mainly integrated into RNA. However, 10 to 20% of AZA is converted by ribonucleotide reductase to its deoxyribose form, thus converting AZA into DAC (Fig. 2). This reduced and triphosphorylated form of AZA is incorporated into genomic DNA and covalently traps DNMT1 at DAC-guanine dinucleotides at the replication fork [15]. Other replication-independent mechanisms have been proposed as well and are reviewed elsewhere [14].

Both AZA and DAC have been thoroughly investigated in clinical trials [16–20] and their clinical efficacy supported through real-world registry data [21–24]. Both are approved for the treatment of MDS, AML and CMML (Table 1). Current National Cancer Center Network (NCCN) guidelines recommend both AZA and DAC as front-line treatment for elderly patients with MDS, CMML or AML who are ineligible for allogeneic stem cell transplantation [25, 26]. Current clinical trials are testing AZA and DAC in various solid tumors, mainly as drug combination partners (Table 2).

**Table 1:** Approval status of hypomethylating agents (HMAs)

	AZA		DAC	
	FDA	EMA	FDA	EMA
MDS				
- low-risk	Yes^a^ (19.05.2004)	No	Yes^c^(02.05.2006)	No
- high risk	Yes^a^ (19.05.2004)	Yes^b^ (17.12.2008)	Yes^c^ (02.05.2006)	No
CMML-FAB				
- CMML-MD	Yes (19.05.2004)	Yes^b^ (17.12.2008)	Yes^c^(02.05.2006)	No
- CMML-MP	Yes (19.05.2004)	No	Yes^c^ (02.05.2006)	No
CMML-WHO				
- CMML-I	Yes (19.05.2004)	No	Yes^c^(02.05.2006)	No
- CMML-II	Yes (19.05.2004)	Yes^b^ (17.12.2008)	Yes^c^ (02.05.2006)	No
AML				
- 20–30% BM blasts	Yes (19.05.2004)	Yes^b,d^ (17.12.2008)	Yes^d,e^ (02.05.2006)	Yes^d,e^ (20.09.2012)
> 30% BM blasts	No	Yes (30.10.2015)	No	Yes^d,e^ (20.09.2012)

^a^if accompanied by neutropenia or thrombocytopenia requiring transfusions

^b^not eligible to allo-SCT

^c^including previously treated and untreated de novo and secondary MDS of all FAB-subtypes

^d^AML with 20–30% BM blasts and multilineage dysplasia, formerly RAEB-t

^e^ > 65a, not eligible for intensive CTX, de novo or secondary, newly diagnosed AML

**Table 2:** Current status of clinical trials testing combinations of HMA (epigenetic priming) with strategies targeting checkpoint receptors/ligands

HMA (Synonym) [Application Route]	Checkpoint Target (Synonym)	Compound (Synonym) [Application Route]	Status (design)	Indication	ClinicalTrials.gov identifier
Azacitidine (CC-486) [p.o., 300 mg d1-15]	CD279 (PD1)	Pembrolizumab (Lambrolizumab, MK-3475) [i.v., 200 mg Q3W]	Phase II (non-randomized)	Metastatic melanoma, other skin neoplasms	NCT02816021
Azacitidine (CC-486) [p.o., 300 mg d1-14, Q3W]	CD279 (PD1)	Pembrolizumab (Lambrolizumab, MK-3475) [i.v., 200 mg Q3W, d1]	Phase II (randomized) • MK-3475 + CC-486 • MK-3475 + placebo	Previously treated locally advanced or metastatic NSCLC	NCT02546986
Azacitidine [s.c., 100 mg d1-5, Q4W]	CD279 (PD1)	Pembrolizumab (Lambrolizumab, MK-3475) [i.v., 200 mg Q3W]	Phase II (non-randomized)	Chemo-refractory metastatic CRC	NCT02260440
Azacitidine [s.c./i.v., 75 mg/m2 d1-7 Q4W]	CD279 (PD1)	Pembrolizumab (Lambrolizumab, MK-3475) [200 mg i.v., Q3W, d8]	Phase II (non-randomized)	Relapsed/refractory AML and newly diagnosed AML >65 years	NCT02845297
Azacitidine (CC-486) [p.o., 300 mg d1-14 or d1-21, Q4W]	CD279 (PD1)	Pembrolizumab (Lambrolizumab, MK-3475) [200 mg i.v., Q3W]	Phase II (randomized) • CC-486 100 mg + MK-3475 • CC-486 100 mg BID + MK-3475 • CC-486 300 mg d1-14 + MK-3475 • CC-486 300 mg d1-21 + MK-3475	Platinum resistant ovarian, fallopian tube or primary peritoneal cancer	NCT02900560
Azacitidine (CC-486) [p.o., 300 mg d1-14 or d1-21, Q4W] +/- Romidepsin (HDAC-I) [7 or 14 mg/m2, d1,8,15, Q4W]	CD279 (PD1)	Pembrolizumab (Lambrolizumab, MK-3475) [200 mg i.v., Q4W, d1 + 15]	Phase I (randomized) • CC-486 + MK-3475 • Romidepsin + MK-3475 • CC-486 + Romidepsin + MK-3475	Microsatellite stable advanced CRC	NCT02512172
Guadecitabine (SGI-110) [s.c., d1-4, Q4W]	CD279 (PD1)	Pembrolizumab (Lambrolizumab, MK-3475) [200 mg i.v., Q4W, d5]	Phase II (non-randomized)	Platinum resistant ovarian, fallopian tube or primary peritoneal cancer	NCT02901899
Azacitidine [s.c., 40 mg/m2, d1-6, d8-10, Q4W] +/- Entinostat (HDAC-I) [p.o., 8 mg, d3 + 10, Q4W]	CD279 (PD1)	Nivolumab (BMS-936558, MDX-1106) [i.v., 3 mg/kg, Q2W]	Phase II (randomized, sequential) • 2 cycles AZA + Romidepsin, followed by Nivolumab • Nivolumab	Recurrent metastatic NSCLC	NCT01928576
THU-decitabine [p.o., 10 mg/kg/0.2 mg/kg, d1-2, Q1W]	CD279 (PD1)	Nivolumab (BMS-936558, MDX-1106) [i.v., 3 mg/kg, Q2W]	Phase II (randomized) • 2 cycles AZA + Romidepsin, followed by Nivolumab • Nivolumab	2nd line therapy for NSCLC	NCT02664181
Azacitidine [s.c./i.v., 75 mg/m2 d1-7 Q4W]	CD279 (PD1)	Nivolumab (BMS-936558, MDX-1106) [i.v., 3 mg/kg, Q4W, d1 + 14]	Phase II (non-randomized)	Relapsed/refractory AML and newly diagnosed AML >65 years	NCT02397720
Azacitidine [s.c./i.v., 75 mg/m2 d1-7, Q4W] Lirilimab (anti-KIR mAb) [d7, Q4W]	CD279 (PD1)	Nivolumab (BMS-936558, MDX-1106) [i.v., 3 mg/kg, Q4W, d7 + 21]	Phase II (non-randomized)	High-risk MDS	NCT02599649
Azacitidine [s.c., 75 mg/m2 d1-5, Q4W]	CD279 (PD1) CD152 (CTLA-4)	Nivolumab (BMS-936558, MDX-1106) [i.v., 3 mg/kg, Q3W, d6] Ipilimumab (BMS-73406, MDX-010) [i.v., 3 mg/kg, Q3W, d6]	Phase II (non-randomized, parallel assignment) • AZA + Nivolumab • AZA + Ipilimumab • AZA + Nivolumab + Ipilimumab	Previously untreated MDS	NCT02530463
Decitabine [d1-5, Q4W]	CD152 (CTLA-4)	Ipilimumab (BMS-73406, MDX-010) [i.v., 3 mg/kg, Q8W, d1]	Phase I (non-randomized)	Relapsed/refractory post allo-HSCT MDS RAEB I/II or AML	NCT02890329
SGI-110 [30 mg/m2, d1-5, Q3W]	CD152 (CTLA-4)	Ipilimumab (BMS-73406, MDX-010) [i.v., 3 mg/kg, Q3W]	Phase I (non-randomized)	Unresectable or metastatic melanoma	NCT02608437
Azacitidine [s.c., 75 mg/m2, d1-7, Q4W]	CD152 (CTLA-4) CD274 (PD-L1)	Tremelimumab (CP-675,206) Durvalumab (MEDI-4736)	Phase I (non-randomized) • Tremelimumab + Ipilimumab • AZA + Tremelimumab + Ipilimumab	MDS relapsed/refractory to HMA	NCT02117219
Azacitidine (CC-486) [p.o., 300 mg d1-14, Q4W]	CD274 (PD-L1)	Durvalumab (MEDI-4736) [i.v., 1500 mg, Q4W, d1]	Phase II (non-randomized)	Microsatellite stable CRC, platinum-resistant ovarian cancer, ER+ and HER2- breast cancer	NCT02811497
Azacitidine [s.c., 75 mg/m2, d1-7, Q4W]	CD274 (PD-L1)	Durvalumab (MEDI-4736) [i.v., 1500 mg, Q4W, d1]	Phase II (randomized) • AZA + Durvalumab • AZA	Previously untreated high-risk MDS or AML >65 years ineligible for allo-SCT	NCT02775903
Azacitidine (CC-486) [p.o., 200 mg d1-21, Q4W]	CD274 (PD-L1)	Durvalumab (MEDI-4736) [i.v.]	Phase II (randomized) • CC-486 + Durvalumab • CC-486	MDS refractory to Azacitidine or Decitabine	NCT02281084
Azacitidine [s.c., 75 mg/m2, d1-7 or d1-5 + 8-9, Q4W]	CD274 (PD-L1)	Azetolizumab (MPDL-3280A) (RG7446) [i.v., 840 mg, d1 + 22, Q4W]	Phase II (non-randomized)	HMA naive or relapsed/refractory MDS	NCT02508870
Azacitidine [s.c. or i.v., 75 mg/m2, d1-7, Q4W]	CD274 (PD-L1)	Avelumab (MSB-0010718C) [i.v., 3 mg/kg, d1 = +14, Q4W]	Phase I/II (non-randomized)	Relapsed/refractory AML	NCT02953561

Demethylation of aberrantly methylated CpG-rich gene promoters was initially the central explanation for the anti-tumor activity of HMAs [27–29]. At high doses HMAs are cytotoxic, whereas at low doses HMAs reactivate silenced genes and cellular differentiation [30]. Clinical trials for the treatment of MDS and AML used high cytotoxic doses (several grams per m^2^) of HMAs [31], but subsequently, prolonged repetitive exposure schedules at lower-doses (20 mg/m^2^ for DAC and 75 mg/m^2^ over 7 days for AZA) were found to improve clinical efficacy, with reduced and usually mild non-hematological toxicities [16, 18, 32–36]. Recent investigations into the concentration-dependent effects of demethylation mediated by HMAs on the immune response will be discussed further on.

### Introduction to viral defense mechanisms and interferon (IFN) signaling

Pathogen (e.g. virus) detection in infected cells occurs via pathogen-sensing pattern-recognition receptors (PRRs). PRRs are proteins expressed by cells of the innate immune system to identify pathogen-associated molecular patterns (PAMPs) and damage-associated molecular patterns (DAMPs) [37]. They can be classified into membrane-bound PRRs (including Toll-like receptors (TLRs)), cytoplasmic PRRs (including NOD-like receptors (NLRs), RIG-1-like receptors (RLRs)), and secreted PRRs.

**Fig. 3:**
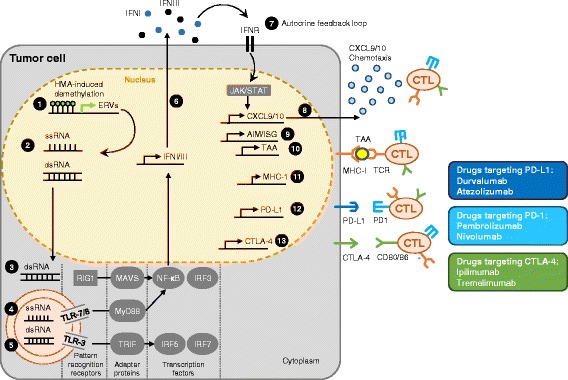
Proposed mechanism of HMA-induced IFN response. The figure shows an epithelial tumor cell where the ERV promoters are methylated. Therapy with AZA/DAC leads to demethylation of ERV promoters (1), resulting in transcription of ERV genes, ssRNA and dsRNA (2). In the cytoplasm, ERV dsRNA is sensed by the pathogen recognition receptor (PRR) RIG1 and MDA5, which activate the transcription factors NFκB and IRF3 after binding to the adapter protein MAVS (3). The endosomal membrane-bound TLR-7 and -8 recognize endosomal ssRNA, and activate the transcription factors NFκB and IRF3 after binding to the adapter molecule MyD88 (4). The endosomal membrane-bound TLR-3 recognizes endosomal dsRNA, and activates the transcription factors IRF-5 and -7 after binding to the adapter molecule TRIF (5). These three pathways all drive the expression and secretion of interferon type 1 and 3 (INFI/III) (6). IFNI and III signal back via an autocrine feedback loop and the INF-receptor (IFNR), which signals via JAK/STAT (7). This results in the up-regulation and secretion of the chemokines CXCL9 and 10, which attract tumor-specific CTLs (8). In addition, AIM and ISGs are upregulated, which also aid in reactivation of dormant anti-tumor immunity (9). Furthermore, TAAs are upregulated (10), as are MHC-I molecules (11), which together enhance the immunologic visibility of the tumor cells and enable them to be recognized by the TCR of tumor-specific CTLs. Treatment with HMAs also results in the unwanted up-regulation of inhibitory immune checkpoint receptors (PD-1, CTLA-4) (12) and their ligands (PD-L1, PD-L2, CD80, CD86) (13), which can result in secondary resistance to HMAs, but may also be exploited as a sensitizing or priming strategy for targeted treatment with immune checkpoint modulators

Detection of viral double-stranded RNA (dsRNA) within the cell occurs via the endosomal membrane-bound TLR-3 receptor. On binding dsRNA, TLR-3 signals through the signal adaptor protein TIR-domain-containing adapter-inducing interferon-β (TRIF) to activate the transcription factors interferon response factor (IRF)-5 and -7, resulting in the expression of type 1 interferons (IFN), mainly IFNβ (Fig. 3 (4, 5)). In contrast, endosomal membrane-bound TLR-7 and -8 detect GU-rich viral single-stranded RNA and signal via the signal adaptor protein myeloid differentiation primary response gene 88 protein to activate the transcription factors nuclear-factor kappa B and IRF-3 and -7, resulting in the expression of proinflammatory cytokines such as TNFα, IL-1 and IL-12 [38–40] . The cytosolic RLRs retinoid acid inducible gene 1 (RIG-1) and melanoma differentiation associated gene 5 (MDA5) detect viral dsRNA in the cytosol and utilize the adaptor protein mitochondrial antiviral signaling protein (MAVS) to activate downstream signaling via the activation of the transcription factors IRF-3 and -7 and NFκB to induce IFN-I and IFN-III [41–44] (Fig. 3 (3)). Thus, viral infection leads to the production and release of proinflammatory cytokines and IFN-I and -III, which in turn alerts both neighboring cells, as well as cells of the innate and adaptive immune system, and also activates intracellular antimicrobial programs via an autocrine feedback loop (Fig. 3 (6)).

Type I IFNs (eg. IFNα and β that bind to IFNα-receptor (IFNAR)) are expressed as a first line of defense against viral infections, play a central role in the regulation of innate immunity to limit viral spread during the first days of infection, and also activate multifaceted antitumor immunity. Type 2 IFN (IFNγ, binds to the IFNγ-receptor (IFNGR)) also displays some of the anti-viral and anti-tumoral properties of type 1 IFNs and potentiates their effects, but predominantly stimulates the adaptive immune system, primarily T-cells [45]. Type 3 IFNs include IFNλ1, λ2 and λ3 (also known as interleukin (IL) 29, IL-28A, and IL-28B, respectively) which signal through a heterodimeric signaling complex composed of IL10R2 and IL28RA and induce a type 1 IFN-like response, and are likewise induced by viral infections [45, 46].

On binding to their respective membrane-bound receptor, IFNs induce the Janus kinase (JAK)/signal transducer and activator of transcription (STAT) signaling, activating transcription of so-called IFN-stimulated genes (ISGs) (Fig. 3 (6,8)). This process is also regulated by epigenetic mechanisms, such as microRNAs that suppress STAT1 expression or chromatin remodeling processes required to initiate transcription of ISGs [45, 47]. ISGs activate intracellular antimicrobial programs, stall the expression of viral genes, can degrade viral nucleic acids, and importantly inhibit cellular proliferation. These events contribute to the containment of viral spread [48] and are also associated with anticancer immunity [49] (Fig. 3 (7-10)).

### Introduction to retrotransposons and endogenous retroviruses (ERVs)

**Fig. 4:**
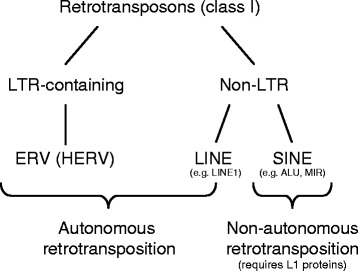
Taxonomy of retrotransposons. The so-called retrotransposons or class I transposons as opposed to class II (DNA) transposons (not depicted) can be grouped into long terminal repeat (LTR) containing and non-LTR transposons. The best investigated LTR retrotransposons are the human endogenous retroviral elements (ERV). Together with the non-LTR retrotransposons LINE (long interspersed nuclear elements), human ERVs are capable of retrotransposition in an autonomous manner. In contrast, short interspersed nuclear elements (SINEs) like ALU or MIR (mammalian-wide interspersed repeats) sequences cannot perform autonomous retrotransposition. Nevertheless, ALU sequences may be able to move with the help of active LINE elements

Around 45% of the human genome is composed of sequences derived from transposable elements [50]. Transposons are DNA sequences able to change their position within the genome (i.e. move from one part to another). There are two categories: Class I transposons (~42% of genome) are referred to as retrotransposons and require RNA intermediates and reverse transcription,whereas Class II transposons (~2–3% of the genome) move via DNA intermediates. In brief, class I retrotransposons can be grouped into long terminal repeat (LTR) and non-LTR retrotransposons (Fig. 4). Non-LTR retrotransposons consist of two subtypes, long interspersed elements (LINEs) [51] and short interspersed elements (SINEs) [52] (Fig. 4). The most common LINEs are LINE-1 and LINE-2, and the most common SINEs are Alu-elements and mammalian wide interspersed repeats (MIR) Fig. 4). The largest group of LTR-containing retrotransposons are endogenous retrovirus transposons (ERVs) and constitute ~8% of the human genome [53] Fig. 4). Full-length ERVs contain LTRs that flank non-repetitive sequences. The non-repetitive sequences contain several protein-coding sequences necessary for transcription, reverse transcription, and integration of the viral genome as well as sequences coding for viral envelope proteins (Gag, Pol and Env). ERVs together with LINEs are autonomously capable of retrotransposition, whereas SINEs do not encode a functional reverse transcriptase and require the LINE machinery, thus functioning as non-autonomous retro-elements (Fig. 4).

The abundance of endogenous ERVs in the human genome can be explained by the integration of exogenous retroviruses that have infected germ-line cells and integrated viral DNA into the human genome [54–56] [57]. Most of these retroviral insertions are evolutionarily ancient, and have been inactivated by mutation and disintegration of the viral genome, so are considered to be ‘junk’ DNA with no function. Some ERVs are, however, able to be transcribed and reintegrated into the host genome [58]. These elements play relevant roles in shaping the genome, gene expression and regulation [59], and cell fusion processes during placentogenesis and embryogenesis [60–62]. Furthermore, LTR-containing ERVs may act as alternate promoters or enhancers that result in tissue-specific gene expression [53, 63]. This observation is of particular interest with respect to the recent discovery that gene regulatory networks have evolved through co-option of endogenous ERV regulatory sequences [64–66]. ERV-derived regulatory sequences within a network share common tissue-specific epigenetic makeups [67] and this might explain concerted reactivation upon epigenetic modulation. It has also been shown that non-LTR retrotransposons can be incorporated into novel genes and evolve new functionality [68, 69]. Interestingly, it was recently found that specific LINE-1 retrotransposons in the human genome are actively transcribed and that the associated LINE-1 RNAs are tightly bound to nucleosomes and are essential in the establishment of the local chromatin environment [70]. However, during adulthood such mobile elements are silenced primarily via CpG methylation [71]. For example, LINE-1 retrotransposons retain ~80–100 copies throughout the human genome that remain capable of retrotransposition, but are epigenetically silenced in normal cells. LINE-1 demethylation has thus been used as a control measure for the induction of global hypomethylation by HMAs in a given experimental setting [72–74].

Both LINE-1 and ERVs have been associated with tumorigenesis, and somatic insertions of these transposons have been found to confer a selective growth advantage to tumor cells [75, 76]. It has also been suggested that younger ERVs (i.e. more recently integrated ERVs) may play a role in human diseases including neurologic diseases (reviewed in [77]) and cancer [78]. ERVs may not only be directly disease causing, but may also modulate immunity, and evidence exists indicating a general role for ERVs in the regulation of interferon (gamma) responses [79].

## HMAs (RE)Induce expression of genes associated with antitumor immune responses

### Tumor associated antigens (TAAs)

Several reports have described an upregulation of TAAs by AZA in MDS and AML cells, such as the cancer-testis antigen (CTA) and New York Esophageal Squamous Cell Carcinoma-1 antigen [80, 81]. This is in-line with observations of AZA effects in other malignancies [82–84] and is attributable to demethylation of hypermethylated CpG islands located at gene promoters [85]. The upregulation of TAA expression resulted in an increased induction of tumor-specific cytotoxic T-lymphocytes (CTLs) in 15 MDS and AML patients treated with AZA and the HDAC-inhibitor valproate sodium [86]. Of clinical interest, 8/11 patients with a documented TAA-specific CTL response achieved a major clinical response to AZA, including 4 patients with complete remission. Induction of TAA-specific CTL response also correlated temporally with a reduction in the percentage of bone marrow blasts [86].

Increased TAA expression induced by AZA might also be partly supported by improved TAA presentation on the cell surface to CTLs, as data from solid malignancies suggest that AZA can lead to increased HLA class I expression [87]. Treatment of AML cell lines in vitro with DAC in combination with the HDAC-inhibitor chidamide increased the expression of preferentially expressed antigen of melanoma (PRAME), a known TAA in AML. Pretreatment of AML cells with DAC and/or chidamide led to increased killing by PRAME-specific CTLs in vitro [88].

### The AZA immune gene set (AIM)

A series of recent studies have aimed to investigate the effects of low-dose HMAs (<500nM) on immune regulation and alterations in the immune response in the setting of (mainly) epithelial tumors [89–93]. Initial transient exposure of cancer cell lines to HMAs (24 h or 72 h), followed by cultivation in the absence of HMAs has given new insights into the mechanisms of HMA-mediated anti-tumor effects. Tsai et al. demonstrated that transient exposure of AML and breast cancer cell lines to DAC and AZA induces delayed (with respect to drug removal from cell culture), prolonged gene promoter demethylation; and sustained changes in gene expression [89]. Transcriptional changes included the up regulation of several central TSGs (such as cyclin dependent kinase inhibitor *1A*, *1C*, *2A*, *2B*; and alternate reading frame protein p14) [89]. These transcriptome and methylome changes were accompanied by reduced tumorigenicity and self-renewal capacities in both cell lines and primary samples from AML and breast cancer patients [89]. Such time-delayed, sustained responses to HMAs at the molecular level provides a possible explanation for why most patients require 3–6 treatment cycles before achieving a clinical response, and why continuous treatment every 4 weeks is necessary to sustain these responses [16–21, 23, 24].

Other groups have analyzed mRNA expression and DNA methylation profiles upon low-dose AZA treatment of several solid tumor cell lines, including breast-, colorectal-, ovarian- and non-small cell lung cancer [90, 91]. Li et al. defined an ‘AZA immune gene set’ that is comprised of 317 genes that were at least two-fold upregulated after AZA treatment [91]. This ‘AZA immune gene set’ includes genes associated with IFN and cytokine signaling, antigen presentation, and inflammation [91]. Furthermore, analyzing gene expression data from the cancer genome atlas (TCGA) and the gene expression omnibus revealed that the ‘AZA immune gene set’ can cluster several solid tumor types including ovarian, breast, colorectal, non-small cell lung cancer and melanoma – into low and high expressing cancer subtypes [91]. These in vitro observations could also be recapitulated in primary tumor samples from patients with triple -negative breast cancer (NCT01349959) or colorectal cancer (NCT01105377). In these studies, combination treatment with AZA and the HDAC-inhibitor entinostat led to an upregulation of the ‘AZA immune gene set’. This upregulation could still be observed in a biopsy taken 6 months after initiation of therapy in one breast cancer patient [91, 94].

The expression of C-X-C motif chemokine ligands (CXCL) 9 and 10 in ovarian and colon cancer cell lines has been shown to be regulated by epigenetic enzymes, including the histone methyltransferase enhancer of zeste 2 polycomb repressive complex 2 and DNMT1 [95, 96]. Both chemokines are within the AZA immune gene set and are upregulated in response to AZA treatment. DAC has also been shown to induce expression of CXCL9 and 10 in several epithelial cancer cell lines and in primary ovarian cancer cells [91, 95]. CXCL9 and −10 have also been reported to attract tumor-infiltrating lymphocytes and immunological infiltrates, positively linked with better clinical outcomes in human serous ovarian cancer [95, 97–99].

Taken together these in vitro and in vivo investigations demonstrate that upregulation of immunomodulatory pathways induced by low-dose AZA treatment, may reverse an immune-evasion phenotype and subsequently may (re)sensitize the tumor for immunotherapy [90, 91].

### Endogenous retroviral elements (ERVs)

As discussed, the ‘AZA immune gene set’ includes genes that are associated with interferon signaling and that participate in immune responses to viral infections. These include viral response genes (such as TLR-3, MDA5, RIG-1, MAVS, IRFs, NFκB and ISGs), with important roles in the detection and abrogation of viral infections and establishing effective antitumor immunity [47, 100]. Interestingly, some human tumors have been reported to exhibit elevated ERV transcript levels [101–103]. In one study, primary ovarian tumor samples from 19 patients showed a high correlation between ERV transcript levels and the expression of viral defense genes (*p* < 0.0001) [92], indicating that ERV transcript upregulation was accompanied by a viral defense gene expression signature.

Recently, Chiappinelli et al. and Roulois et al. uncovered a new molecular mechanism of action of transient low-dose treatment of tumor cell lines with HMAs. The authors showed that global hypomethylation was accompanied by the demethylation of ERV sequences [92, 93]. The observed increase (up to several thousand-fold over control cells) of dsRNA viral transcripts in the cytoplasm of the cancer cells activated innate PRRs, as well as transcription factor IRF-7, resulting in the induction and secretion of IFN-I/III [92, 93]. As discussed above, these IFNs signal back (in an auto- and paracrine manner) and via activation of STATs induce the transcription of ISGs that mediate anti-tumor effects. This HMA-induced upregulation of ERV transcripts has been termed ‘viral mimicry’ and may result in the induction of effective anti-tumor immunity.

Chiappinelli et al. reported that low-dose AZA treatment of human ovarian cancer cell lines led to demethylation of the *ERV-Fc2* gene promoter, with subsequent upregulation of intracellular dsRNA transcripts of the viral envelope genes *Fc2* and *syncytin-1* [92]. Furthermore, the authors showed that both AZA and DAC increased the expression of several other ERV transcripts [92]. Following HMA withdrawal, activation of ERVs peaked at day 7 and resulted in the upregulation of several viral defense genes including IFNγ-inducible protein 16 (IFI16), IFN-induced protein 44 (IFI44) and IFN-induced protein 44-like (IFI44L), in an IFNβ- and JAK/STAT-dependent manner. This confirmed that AZA induces an IFN type 1 response with subsequent upregulation of ISGs [92].

Similar observations were made in colorectal cancer cell lines by Roulois et al. The authors showed that transient low-dose treatment (0.3 μM) with DAC, followed by cultivation for 42 days without the drug, resulted in two distinct groups of gene expression-change patterns: early and late response genes. Early response genes were defined as genes whose expression level changed within 5 days of DAC treatment [93], and subsequently returned to baseline levels after 37 days. In contrast, late-response genes showed significant upregulation that peaked 24 days after DAC treatment and was sustained for a further 18 days. The late-response group was enriched in genes required for the innate RNA-sensing pathway and IFN response signaling components [93]. Furthermore, the IFN type 3 receptor genes IL29 and IL28a and several ISGs were induced by low-dose DAC treatment in a JAK/STAT dependent manner [93]. Further analysis of the late-response genes revealed that the majority were direct targets of the IRF7 transcription factor. Knock-down of IRF7 and/or targeting of the cytosolic RNA sensing pathway (RIG-1, MDA5 and MAVS) by short hairpin (sh)RNAs was sufficient to block DAC-induced upregulation of IFN response genes. Furthermore, knock-down of MAVS also abolished the observed DAC-mediated reduction in frequency of cancer-initiating cells in colorectal cancer cell lines and in primary colorectal cancer cells [93].

Since MDA5 recognizes dsRNAs of viral origin [39], the authors investigated whether DAC upregulates dsRNA expression. The colorectal cancer cell line LIM1215 showed an increase in cytosolic dsRNA expression upon treatment with DAC, and RT-PCR revealed a strong increase in 10 selected ERV transcripts [93]. These experiments showed for the first time that transient low-dose DAC treatment of colorectal cancer cells induces a type 3 IFN response via the induction of dsERV transcripts [93], which in turn induces apoptosis and reduces cellular proliferative capacity. In this seminal work the authors showed that the diminishing effect of DAC on the growth and self-renewal capacity of colorectal cancer cells is very much dependent on DAC-induced upregulation of viral dsRNAs. This upregulation activates the MDA5/MAVS/IRF7 pathway and subsequently induces an interferon response [93]. All the above indicates that the MDA5/MAVS/IRF7 signaling pathway is a novel therapeutic target in (colorectal) cancer.

As discussed above (section B: The AZA immune gene set (AIM)), cancer samples from the TCGA (melanoma, ovarian, colorectal, breast and lung) could be clustered into high and low immune groups according to the levels of AZA-induced expression of IFN viral defense genes (*IRF7*, *IFI27*, *RIG-1*, *IFI44*, *IFI44L*, *IFI16*, *STAT1*, *IFNB1*, *DDX41*, *MX1*, *OASL*, *TMEM173*, *MB21D1*, *IFI6*) [91, 92]. This is compelling when considering other studies showing that high expression of the viral defense gene signature appears to correlate with improved response and long-term benefit in patients with melanoma when treated with immune checkpoint inhibitors ipilimumab or tremelimumab. Both ipilimumab and tremelimumab target cytotoxic T lymphocyte associated molecule 4 (CTLA-4) and activate CTLs [92, 104]. Therefore, as HMAs have been shown to induce both ERVs and viral defense genes, we hypothesize that these drugs may be able to alter oncogenic signaling circuitry in several ways that may render tumor cells more susceptible to immune therapy .

The discussed work on new molecular mechanisms of HMA demonstrates the induction of ERV transcripts, the upregulation of genes involved in effective antitumor immunity, and the induction of IFNI/III responses in a wide variety of solid and hematologic cancers. This greatly extends the possible therapeutic rationale for the use of HMAs in solid tumors. However, it has to be mentioned that the reactivation of ERVs by HMA treatment might increase genomic instability, resulting in acquisition of new mutations, disease progression, immune evasion, and development of drug resistance [105].

## HMAs (RE)Induce expression of genes associated with tumor immune evasion

### Inhibitory immune checkpoint receptors

Immune checkpoint blockade therapy has gained considerable attention in recent years. Different monoclonal antibodies targeting CTLA-4, programmed death receptor 1 (PD-1) or programmed death ligand 1 (PD-L1) have been FDA approved in metastatic melanoma, advanced metastatic non-small cell lung cancer, renal cell carcinoma and urothelial carcinoma [106]. Although these therapies have been very successful in a large proportion of patients, there remain a number of patients who do not respond to immune checkpoint blockade therapy [107–109].

There is an increasing body of evidence explaining resistance mechanisms, with the tumor microenvironment thought to be key to primary and/or secondary resistance to therapeutic immune checkpoint modulators [106]. Factors that contribute to primary resistance to immune checkpoint blockade therapy are: low numbers of tumor infiltrating lymphocytes; epigenetic silencing of chemokines; type one immunity (T-helper 1 mediated-immunity); and low expression of specific immune signaling molecules like PD-L1, type 1 IFN, and major histocompatibility complex (MHC) 1 molecules [106].

It has been noted that successful anti-tumor T-cell priming requires a critical number of tumor infiltrating type 1 IFN-producing dendritic cells [110, 111]. It was recently shown that facilitating T-cell infiltration into the tumor microenvironment, by targeting the tumor necrosis factor superfamily member LIGHT (also known as TNFSF14, tumor necrosis factor superfamily member 14), can overcome resistance to PD-L1 blockade therapy in a xenograft mouse model of colon cancer and fibrosarcoma [112]. Furthermore, activation of type 1 IFN responses in murine melanomas with low numbers of tumor-infiltrating lymphocytes was associated with prolonged survival in PD-L1 immune-checkpoint blockade therapy [113].

Yang et al. investigated the expression of PD-1, PD-L1, PD-L2, PD-1 and CTLA-4 after HMA treatment in 124 patients with MDS, AML and CMML [114]. An increase in HMA-induced expression of these checkpoint molecules was observed and correlated with dose-dependent (partial) promoter demethylation. The authors therefore proposed that checkpoint gene reactivation may be more dependent on demethylation level than on baseline methylation level [114]. Upregulation of molecules of the PD/PD-L axis as well as CTLA-4 was associated with resistance to HMA treatment, disease progression, and shorter overall survival (OS). This observation is likely due to T-cell exhaustion and resulting tumor immune evasion [114]. Similar results were also reported in another study by Orskov et al. AZA treatment of 27 patients with MDS, AML and CMML resulted in the upregulation of PD-1 in peripheral blood T-cells of patients with MDS; and this occurred via PD-1 promoter demethylation [115]. Of note, patients that did not show PD-1 promoter demethylation after HMA treatment had a better objective response rate and OS [115].

Upregulation of inhibitory checkpoint molecules due to HMA-induced demethylation is an unwanted side-effect that can result in drug resistance and loss of response. However, this could be therapeutically exploited, as it may render tumor cells susceptible to immune checkpoint blockade therapy. This is an interesting and promising therapeutic strategy that is currently being tested in clinical trials (Table 2). Further details on this topic are reviewed by Greil et al. [116].

### Ligands for inhibitory immune checkpoint receptors

CD80 and CD86 are usually present on antigen presenting cells and act as ligands for both the activating immune checkpoint receptor CD28 and inhibitory checkpoint receptor CTLA-4. The affinity and avidity are greater for CTLA-4 enabling it to outcompete CD28 for its ligands [117].

DAC has been shown to induce tumor-specific CTLs in a murine tumor model via upregulation of CD80 on the thymoma cell line EL4 [118], resulting in enhanced immunological co-stimulation via CD80, increased CTL infiltration of tumors, and ultimately tumor rejection after DAC treatment of mice [118]. HMAs have also been shown to induce the expression of the co-stimulatory molecule CD86 on AML cells, which was assumed to be responsible for increased CTL-mediated killing of AML cells [88]. Therefore HMAs not only increase the ‘immunologic visibility’ of the target cells for CTLs, leading to more effective CTL killing, but also activate more tumor-specific CTLs.

## HMAs as sensitizers of immune checkpoint modulators

HMA-induced upregulation of inhibitory immune checkpoint molecules on malignant cells and T-cells could be exploited to prime or (re)sensitize cancer cells with primary resistance to immune checkpoint blocking therapies. Recent work has demonstrated that combinatorial treatment with anti-CTLA-4 antibodies and low-dose AZA or DAC results in significantly decreased tumor growth of melanoma cells in a murine xenograft setting, compared with CTLA-4 therapy alone [92]. This preclinical rationale supports exploring HMAs as combination partners to prime or sensitize patients to immune-checkpoint blockade therapy in clinical trials.

Several clinical trials testing various combinations of HMAs with checkpoint modulators are currently being planned or are under way (summarized in Table 2). Within these trials it will be of important to define predictive biomarkers to identify patients who will benefit the most from such combination regimens and to further define the role of HMAs as ‘checkpoint-inhibitor sensitizers’. It should also be addressed whether, and to what extent, HMAs may induce ERV expression in non-malignant cells and whether this influences side-effects and/or toxicity. Additionally, it will be of considerable interest to investigate whether LINEs also contribute to the HMA-induced increase of dsRNA species in the cytosol of malignant and/or non-malignant cells. Future genome/epigenome-wide investigations into the molecular mechanism of epigenetic therapies should consider viral repetitive sequences in their analysis.

Another line of investigation is the effect of vitamin C administration on the efficacy of HMAs. Recently, vitamin C was reported to augment the induction of ERVs and the induction of viral defense pathways by DAC in in vitro models of human colon, breast, and hepatocellular carcinoma, as well as AML [119]. In immune checkpoint therapy, many cancer patients are deficient in vitamin C; therefore, incorporation of vitamin C into treatment protocols may further increase the clinical efficacy of HMAs.

## Conclusions

HMAs were initially synthesized in the 1960s, and since then their effects on mammalian cells as well as their clinical applicability have been explored considerably [120]. The main mechanism of action thought to be central to the anti-tumor effects of AZA and DAC is the reactivation of aberrantly silenced TSGs and subsequent induction of apoptosis or differentiation, both hindering tumor cell viability. This review has discussed new evidence that suggests a novel mode of action, where HMAs influence tumor interaction with the host immune system. However, HMAs represent a double-edged sword because HMA-induced up-regulation of immune checkpoint molecules during therapy could reduce immunogenicity of the tumor and can also explain resistance arising during therapy.

HMAs exert several immunological effects: (a) HMA-induced IFN signaling blocks proliferation and lowers the apoptotic threshold of cancer cells [92]; (b) low-dose treatment with HMAs promotes expression of genes that are deregulated in tumors allowing immune evasion (MHC class I, cancer testis antigens, IFN type 1 and 3, ISGs) [90–93]; (c) HMAs induce secretion of CXCL-9 and -10 with subsequent recruitment of lymphocytes to the tumor site and thus increase the immunological visibility of the tumor [95, 121].

Finally, the data discussed in this review strongly imply that HMAs may have the potential to counteract factors that contribute to primary resistance to immune checkpoint blockade therapy, and thus may (re)sensitize tumors with (a) low numbers of tumor infiltrating T-cells, (b) low expression of the IFN-response gene expression signature, and/or (c) high expression levels of inhibitory immune checkpoint molecules to targeted immune checkpoint modulation.

## Abbreviations

AML: Acute myeloid leukemia
AZA: 5-Azacytidine
CMML: Chronic myelomonocytic leukemia
CTLA-4: Cytotoxic T lymphocyte associated molecule 4
CXCL: C-X-C motif chemokine ligand
DAC: 2′-deoxy-5-azacytidine
DDX41: DEAD-box helicase 41
DNA: Deoxyribonucleic acid
DNMT: DNA methyltransferase
EMA: European Medicines Agency
ERV: Endogenous retroviral element
FDA: Food and Drug Administration
HDAC: Histone deacetylase
HMA: Hypomethylating agents
IFI: Interferon induced protein
IFI44L: Interferon induced protein 44 like
IFI6: Interferon alpha inducible protein 6
IFN-b: Interferon beta
IRF: Interferon response factor
ISG: Interferon-stimulated gene
ISGF3: Interferon-stimulated gene factor 3
JAK: Janus kinase
MAVS: Mitochondrial antiviral-signaling protein
MB21D1: Mab-21 domain containing1
MDA5: Melanoma differentiation associated gene 5
MDS: Myelodysplastic syndrome
MHC: Major histocompatibility complex
MX1: MX dynamin like GTPase 1
OASL: 2′-5′-oligoadenylate synthethase-like
OS: Overall survival
PD-1: Programmed death 1
PD-L1: Programmed death-ligand 1
PRR: Pattern recognition receptors
RIG-1: Retinoid acid inducible gene 1
STAT: Signal transducer and activator of transcription
TLR: Toll-like receptor
TMEM173: Transmembrane protein 173
TSG: Tumor suppressor gene

## References

[1] Jones PA (2012). Functions of DNA methylation: islands, start sites, gene bodies and beyond. Nat Rev Genet.

[2] Lister R, Ecker JR (2009). Finding the fifth base: genome-wide sequencing of cytosine methylation. Genome Res.

[3] Baylin SB, Jones PA (2011). A decade of exploring the cancer epigenome - biological and translational implications. Nat Rev Cancer.

[4] Torres IO, Fujimori DG (2015). Functional coupling between writers, erasers and readers of histone and DNA methylation. Curr Opin Struct Biol.

[5] Sharma S, Kelly TK, Jones PA (2010). Epigenetics in cancer. Carcinogenesis.

[6] Jones PA, Baylin SB (2002). The fundamental role of epigenetic events in cancer. Nat Rev Genet.

[7] Eden A, Gaudet F, Waghmare A, Jaenisch R (2003). Chromosomal instability and tumors promoted by DNA hypomethylation. Science.

[8] Baylin SB (2005). DNA methylation and gene silencing in cancer. Nat Clin Pract Oncol.

[9] Jankowska AM, Millward CL, Caldwell CW (2015). The potential of DNA modifications as biomarkers and therapeutic targets in oncology. Expert Rev Mol Diagn.

[10] Khakpour G, Pooladi A, Izadi P, Noruzinia M, Tavakkoly Bazzaz J (2015). DNA methylation as a promising landscape: A simple blood test for breast cancer prediction. Tumour Biol.

[11] Ansari J, Shackelford RE, El-Osta H (2016). Epigenetics in non-small cell lung cancer: from basics to therapeutics. Transl Lung Cancer Res.

[12] Figueroa ME (2010). DNA methylation signatures identify biologically distinct subtypes in acute myeloid leukemia. Cancer Cell.

[13] Bullinger L (2010). Quantitative DNA methylation predicts survival in adult acute myeloid leukemia. Blood.

[14] Pleyer L, Greil R (2015). Digging deep into “dirty” drugs - modulation of the methylation machinery. Drug Metab Rev.

[15] Santi DV, Norment A, Garrett CE (1984). Covalent bond formation between a DNA-cytosine methyltransferase and DNA containing 5-azacytosine. Proc Natl Acad Sci U S A.

[16] Fenaux P (2009). Efficacy of azacitidine compared with that of conventional care regimens in the treatment of higher-risk myelodysplastic syndromes: a randomised, open-label, phase III study. Lancet Oncol.

[17] Fenaux P (2010). Azacitidine prolongs overall survival compared with conventional care regimens in elderly patients with low bone marrow blast count acute myeloid leukemia. J Clin Oncol.

[18] Dombret H (2015). International phase 3 study of azacitidine vs conventional care regimens in older patients with newly diagnosed AML with >30% blasts. Blood.

[19] Kantarjian HM (2012). Multicenter, randomized, open-label, phase III trial of decitabine versus patient choice, with physician advice, of either supportive care or low-dose cytarabine for the treatment of older patients with newly diagnosed acute myeloid leukemia. J Clin Oncol.

[20] Garcia-Manero G (2013). Randomized open-label phase II study of decitabine in patients with low- or intermediate-risk myelodysplastic syndromes. J Clin Oncol.

[21] Pleyer L (2013). Azacitidine in patients with WHO-defined AML - results of 155 patients from the Austrian Azacitidine Registry of the AGMT-Study Group. J Hematol Oncol.

[22] Pleyer L (2014). Azacitidine in 302 patients with WHO-defined acute myeloid leukemia: results from the Austrian Azacitidine Registry of the AGMT-Study Group. Ann Hematol.

[23] Pleyer L (2014). Azacitidine in CMML: matched-pair analyses of daily-life patients reveal modest effects on clinical course and survival. Leuk Res.

[24] Pleyer L (2016). Azacitidine front-line in 339 patients with myelodysplastic syndromes and acute myeloid leukaemia: comparison of French-American-British and World Health Organization classifications. J Hematol Oncol.

[25] National Comprehensive Cancer Network. Clinical Practice Guidelines in Oncology: acute myeloid leukemia guidelines version 2. 2016.

[26] National Comprehensive Cancer Network (2016). Clinical Practice Guidelines in Oncology: myelodysplastic syndromes guidelines.

[27] Bender CM, Pao MM, Jones PA (1998). Inhibition of DNA methylation by 5-aza-2′-deoxycytidine suppresses the growth of human tumor cell lines. Cancer Res.

[28] Cameron EE, Bachman KE, Myohanen S, Herman JG, Baylin SB (1999). Synergy of demethylation and histone deacetylase inhibition in the re-expression of genes silenced in cancer. Nat Genet.

[29] Karpf AR, Jones DA (2002). Reactivating the expression of methylation silenced genes in human cancer. Oncogene.

[30] Jones PA, Taylor SM (1980). Cellular differentiation, cytidine analogs and DNA methylation. Cell.

[31] Santini V, Kantarjian HM, Issa JP (2001). Changes in DNA methylation in neoplasia: pathophysiology and therapeutic implications. Ann Intern Med.

[32] Issa JP (2004). Phase 1 study of low-dose prolonged exposure schedules of the hypomethylating agent 5-aza-2′-deoxycytidine (decitabine) in hematopoietic malignancies. Blood.

[33] Kantarjian H (2007). Results of a randomized study of 3 schedules of low-dose decitabine in higher-risk myelodysplastic syndrome and chronic myelomonocytic leukemia. Blood.

[34] Kantarjian H (2006). Decitabine improves patient outcomes in myelodysplastic syndromes: results of a phase III randomized study. Cancer.

[35] Lubbert M (2001). Cytogenetic responses in high-risk myelodysplastic syndrome following low-dose treatment with the DNA methylation inhibitor 5-aza-2′-deoxycytidine. Br J Haematol.

[36] Wijermans P (2000). Low-dose 5-aza-2′-deoxycytidine, a DNA hypomethylating agent, for the treatment of high-risk myelodysplastic syndrome: a multicenter phase II study in elderly patients. J Clin Oncol.

[37] Cui J, Chen Y, Wang HY, Wang RF (2014). Mechanisms and pathways of innate immune activation and regulation in health and cancer. Hum Vaccin Immunother.

[38] Kawai T, Akira S (2010). The role of pattern-recognition receptors in innate immunity: update on Toll-like receptors. Nat Immunol.

[39] Pichlmair A (2009). Activation of MDA5 requires higher-order RNA structures generated during virus infection. J Virol.

[40] Heil F (2004). Species-specific recognition of single-stranded RNA via toll-like receptor 7 and 8. Science.

[41] Goubau D, Deddouche S, Reis e Sousa C (2013). Cytosolic sensing of viruses. Immunity.

[42] Seth RB, Sun L, Ea CK, Chen ZJ (2005). Identification and characterization of MAVS, a mitochondrial antiviral signaling protein that activates NF-kappaB and IRF 3. Cell.

[43] Barbalat R, Ewald SE, Mouchess ML, Barton GM (2011). Nucleic acid recognition by the innate immune system. Annu Rev Immunol.

[44] Sun Q (2006). The specific and essential role of MAVS in antiviral innate immune responses. Immunity.

[45] Zhou Z (2007). Type III interferon (IFN) induces a type I IFN-like response in a restricted subset of cells through signaling pathways involving both the Jak-STAT pathway and the mitogen-activated protein kinases. J Virol.

[46] Donnelly RP, Kotenko SV (2010). Interferon-lambda: a new addition to an old family. J Interferon Cytokine Res.

[47] Ivashkiv LB, Donlin LT (2014). Regulation of type I interferon responses. Nat Rev Immunol.

[48] Schoggins JW, Rice CM (2011). Interferon-stimulated genes and their antiviral effector functions. Curr Opin Virol.

[49] Zitvogel L, Galluzzi L, Kepp O, Smyth MJ, Kroemer G (2015). Type I interferons in anticancer immunity. Nat Rev Immunol.

[50] Lander ES (2001). Initial sequencing and analysis of the human genome. Nature.

[51] Doucet AJ, et al. Characterization of LINE-1 ribonucleoprotein particles. PLoS Genet. 2010; 6. doi:10.1371/journal.pgen.1001150. 10.1371/journal.pgen.1001150 PMC2951350 20949108

[52] Cordaux R, Batzer MA (2009). The impact of retrotransposons on human genome evolution. Nat Rev Genet.

[53] McCarthy EM, McDonald JF (2004). Long terminal repeat retrotransposons of Mus musculus. Genome Biol.

[54] Kim FJ, Battini JL, Manel N, Sitbon M (2004). Emergence of vertebrate retroviruses and envelope capture. Virology.

[55] Wicker T (2007). A unified classification system for eukaryotic transposable elements. Nat Rev Genet.

[56] Khodosevich K, Lebedev Y, Sverdlov E (2002). Endogenous retroviruses and human evolution. Comp Funct Genomics.

[57] Belshaw R (2004). Long-term reinfection of the human genome by endogenous retroviruses. Proc Natl Acad Sci U S A.

[58] Kassiotis G, Stoye JP (2016). Immune responses to endogenous retroelements: taking the bad with the good. Nat Rev Immunol.

[59] Oliver KR, Greene WK (2011). Mobile DNA and the TE-Thrust hypothesis: supporting evidence from the primates. Mob DNA.

[60] Medstrand P (2005). Impact of transposable elements on the evolution of mammalian gene regulation. Cytogenet Genome Res.

[61] Blond JL (2000). An envelope glycoprotein of the human endogenous retrovirus HERV-W is expressed in the human placenta and fuses cells expressing the type D mammalian retrovirus receptor. J Virol.

[62] Dupressoir A (2009). Syncytin-A knockout mice demonstrate the critical role in placentation of a fusogenic, endogenous retrovirus-derived, envelope gene. Proc Natl Acad Sci U S A.

[63] Goke J, Ng HH (2016). CTRL+INSERT: retrotransposons and their contribution to regulation and innovation of the transcriptome. EMBO Rep.

[64] Chuong EB, Elde NC, Feschotte C (2016). Regulatory evolution of innate immunity through co-option of endogenous retroviruses. Science.

[65] Chuong EB, Elde NC, Feschotte C. Regulatory activities of transposable elements: from conflicts to benefits. Nat Rev Genet. 2016. doi:10.1038/nrg.2016.139. 10.1038/nrg.2016.139 PMC5498291 27867194

[66] Lynch VJ (2016). GENETICS. A copy-and-paste gene regulatory network. Science.

[67] Sundaram V (2014). Widespread contribution of transposable elements to the innovation of gene regulatory networks. Genome Res.

[68] Santangelo AM (2007). Ancient exaptation of a CORE-SINE retroposon into a highly conserved mammalian neuronal enhancer of the proopiomelanocortin gene. PLoS Genet.

[69] Liang KH, Yeh CT (2013). A gene expression restriction network mediated by sense and antisense Alu sequences located on protein-coding messenger RNAs. BMC Genomics.

[70] Chueh AC, Northrop EL, Brettingham-Moore KH, Choo KH, Wong LH (2009). LINE retrotransposon RNA is an essential structural and functional epigenetic component of a core neocentromeric chromatin. PLoS Genet.

[71] Rowe HM, Trono D (2011). Dynamic control of endogenous retroviruses during development. Virology.

[72] Nelson HH, Marsit CJ, Kelsey KT (2011). Global methylation in exposure biology and translational medical science. Environ Health Perspect.

[73] Weisenberger DJ (2005). Analysis of repetitive element DNA methylation by MethyLight. Nucleic Acids Res.

[74] Tabish AM (2015). Assessment of Changes in Global DNA Methylation Levels by Pyrosequencing(R) of Repetitive Elements. Methods Mol Biol.

[75] Lee E (2012). Landscape of somatic retrotransposition in human cancers. Science.

[76] Szpakowski S (2009). Loss of epigenetic silencing in tumors preferentially affects primate-specific retroelements. Gene.

[77] Christensen T (2016). Human endogenous retroviruses in neurologic disease. APMIS.

[78] Strick R, Strissel PL, Baylin SB, Chiappinelli KB (2016). Unraveling the molecular pathways of DNA-methylation inhibitors: human endogenous retroviruses induce the innate immune response in tumors. Oncoimmunology.

[79] Platanias LC (2005). Mechanisms of type-I- and type-II-interferon-mediated signalling. Nat Rev Immunol.

[80] Almstedt M (2010). The DNA demethylating agent 5-aza-2′-deoxycytidine induces expression of NY-ESO-1 and other cancer/testis antigens in myeloid leukemia cells. Leuk Res.

[81] Atanackovic D (2011). Cancer-testis antigen expression and its epigenetic modulation in acute myeloid leukemia. Am J Hematol.

[82] Guo ZS (2006). De novo induction of a cancer/testis antigen by 5-aza-2′-deoxycytidine augments adoptive immunotherapy in a murine tumor model. Cancer Res.

[83] Weber J (1994). Expression of the MAGE-1 tumor antigen is up-regulated by the demethylating agent 5-aza-2′-deoxycytidine. Cancer Res.

[84] Dubovsky JA (2009). Treatment of chronic lymphocytic leukemia with a hypomethylating agent induces expression of NXF2, an immunogenic cancer testis antigen. Clin Cancer Res.

[85] De Smet C, Lurquin C, Lethe B, Martelange V, Boon T (1999). DNA methylation is the primary silencing mechanism for a set of germ line- and tumor-specific genes with a CpG-rich promoter. Mol Cell Biol.

[86] Goodyear O (2010). Induction of a CD8+ T-cell response to the MAGE cancer testis antigen by combined treatment with azacitidine and sodium valproate in patients with acute myeloid leukemia and myelodysplasia. Blood.

[87] Fonsatti E (2007). Functional up-regulation of human leukocyte antigen class I antigens expression by 5-aza-2′-deoxycytidine in cutaneous melanoma: immunotherapeutic implications. Clin Cancer Res.

[88] Yao Y (2013). Increased PRAME-specific CTL killing of acute myeloid leukemia cells by either a novel histone deacetylase inhibitor chidamide alone or combined treatment with decitabine. PLoS One.

[89] Tsai HC (2012). Transient low doses of DNA-demethylating agents exert durable antitumor effects on hematological and epithelial tumor cells. Cancer Cell.

[90] Wrangle J (2013). Alterations of immune response of Non-Small Cell Lung Cancer with Azacytidine. Oncotarget.

[91] Li H (2014). Immune regulation by low doses of the DNA methyltransferase inhibitor 5-azacitidine in common human epithelial cancers. Oncotarget.

[92] Chiappinelli KB (2015). Inhibiting DNA Methylation Causes an Interferon Response in Cancer via dsRNA Including Endogenous Retroviruses. Cell.

[93] Roulois D (2015). DNA-Demethylating Agents Target Colorectal Cancer Cells by Inducing Viral Mimicry by Endogenous Transcripts. Cell.

[94] Connolly RMZC, Zhang Z, Rudek MA, Jeter SC, Slater S, Powers P, Wolff AC, Fetting J, Brufsky AM, Piekarz R, Ahuja N, Somlo G, Garcia AA, Baylin SB, Davidson NE, Stearns V. A Phase 2 Study Investigating the Safety, Efficacy and Surrogate Biomarkers of Response of 5-Azacitidine (5-AZA) and Entinostat (MS-275) in Patients with Advanced Breast Cancer. AACR Annual Meeting 2013, Washington, DC. 2013 (Abs 4666); 2013.

[95] Peng D (2015). Epigenetic silencing of TH1-type chemokines shapes tumour immunity and immunotherapy. Nature.

[96] Nagarsheth N (2016). PRC2 Epigenetically Silences Th1-Type Chemokines to Suppress Effector T-Cell Trafficking in Colon Cancer. Cancer Res.

[97] Musha H (2005). Selective infiltration of CCR5(+)CXCR3(+) T lymphocytes in human colorectal carcinoma. Int J Cancer.

[98] Son DS, Parl AK, Rice VM, Khabele D (2007). Keratinocyte chemoattractant (KC)/human growth-regulated oncogene (GRO) chemokines and pro-inflammatory chemokine networks in mouse and human ovarian epithelial cancer cells. Cancer Biol Ther.

[99] Ohtani H, Jin Z, Takegawa S, Nakayama T, Yoshie O (2009). Abundant expression of CXCL9 (MIG) by stromal cells that include dendritic cells and accumulation of CXCR3+ T cells in lymphocyte-rich gastric carcinoma. J Pathol.

[100] Parker BS, Rautela J, Hertzog PJ (2016). Antitumour actions of interferons: implications for cancer therapy. Nat Rev Cancer.

[101] Stengel S, Fiebig U, Kurth R, Denner J (2010). Regulation of human endogenous retrovirus-K expression in melanomas by CpG methylation. Genes Chromosomes Cancer.

[102] Laska MJ, Nissen KK, Nexo BA (2013). (Some) cellular mechanisms influencing the transcription of human endogenous retrovirus, HERV-Fc1. PLoS One.

[103] Strissel PL (2012). Reactivation of codogenic endogenous retroviral (ERV) envelope genes in human endometrial carcinoma and prestages: Emergence of new molecular targets. Oncotarget.

[104] Snyder A (2014). Genetic basis for clinical response to CTLA-4 blockade in melanoma. N Engl J Med.

[105] Weiss RA (2016). Human endogenous retroviruses: friend or foe?. APMIS.

[106] Pitt JM (2016). Resistance Mechanisms to Immune-Checkpoint Blockade in Cancer: Tumor-Intrinsic and -Extrinsic Factors. Immunity.

[107] Schadendorf D (2015). Pooled Analysis of Long-Term Survival Data From Phase II and Phase III Trials of Ipilimumab in Unresectable or Metastatic Melanoma. J Clin Oncol.

[108] Royal RE (2010). Phase 2 trial of single agent Ipilimumab (anti-CTLA-4) for locally advanced or metastatic pancreatic adenocarcinoma. J Immunother.

[109] Brahmer JR (2012). Safety and activity of anti-PD-L1 antibody in patients with advanced cancer. N Engl J Med.

[110] Diamond MS (2011). Type I interferon is selectively required by dendritic cells for immune rejection of tumors. J Exp Med.

[111] Fuertes MB (2011). Host type I IFN signals are required for antitumor CD8+ T cell responses through CD8{alpha} + dendritic cells. J Exp Med.

[112] Tang H (2016). Facilitating T Cell Infiltration in Tumor Microenvironment Overcomes Resistance to PD-L1 Blockade. Cancer Cell.

[113] Bald T (2014). Immune cell-poor melanomas benefit from PD-1 blockade after targeted type I IFN activation. Cancer Discov.

[114] Yang H (2014). Expression of PD-L1, PD-L2, PD-1 and CTLA4 in myelodysplastic syndromes is enhanced by treatment with hypomethylating agents. Leukemia.

[115] Orskov AD (2015). Hypomethylation and up-regulation of PD-1 in T cells by azacytidine in MDS/AML patients: A rationale for combined targeting of PD-1 and DNA methylation. Oncotarget.

[116] Greil R, Hutterer E, Hartmann TN, Pleyer L. Reactivation of dormant anti-tumor immunity - A clinical perspective of therapeutic immune checkpoint modulation. Cell Commun Signal. 2016;15:14. 10.1186/s12964-016-0155-9 PMC5244547 28100240

[117] Walunas TL, Bakker CY, Bluestone JA (1996). CTLA-4 ligation blocks CD28-dependent T cell activation. J Exp Med.

[118] Wang LX (2013). Low dose decitabine treatment induces CD80 expression in cancer cells and stimulates tumor specific cytotoxic T lymphocyte responses. PLoS One.

[119] Liu M (2016). Vitamin C increases viral mimicry induced by 5-aza-2′-deoxycytidine. Proc Natl Acad Sci U S A.

[120] Von Hoff DD, Slavik M, Muggia FM (1976). 5-Azacytidine. A new anticancer drug with effectiveness in acute myelogenous leukemia. Ann Intern Med.

[121] Sistigu A (2014). Cancer cell-autonomous contribution of type I interferon signaling to the efficacy of chemotherapy. Nat Med.

